# Analysis of the efficacy and safety of conservative treatment of blunt abdominal trauma in children: retrospective study. Conservative treatment of blunt abdominal trauma in children

**DOI:** 10.1590/0100-6991e-20233429-en

**Published:** 2023-03-13

**Authors:** SARAH CRESTIAN CUNHA, ANTONIO GONÇALVES DE-OLIVEIRA, MARCIO LOPES MIRANDA, MARCIA ALESSANDRA CAVALARO PEREIRA-DA SILVA, PATRÍCIA TRABALLI DE CARVALHO PEGOLO, LUIZ ROBERTO LOPES, JOAQUIM MURRAY BUSTORFF-SILVA

**Affiliations:** 1 - Universidade Estadual de Campinas (UNICAMP), Cirurgia - Campinas - SP - Brasil

**Keywords:** Conservative Treatment, Abdominal Injuries, Trauma, Pediatric Emergency Medicine, Tratamento Conservador, Índices de Gravidade do Trauma, Traumatismos Abdominais

## Abstract

**Introduction:**

in Brazil, trauma is responsible for 40% of deaths in the age group between 5 and 9 years old, and 18% between 1 and 4 years, and bleeding is the leading cause of preventable death in the traumatized child. Conservative management of blunt abdominal trauma with solid organs injury - started in the 60s - is the current world trend, with studies showing survival rates above 90%. The objective was to assess the efficacy and safety of conservative treatment in children with blunt abdominal trauma treated at the Clinical Hospital of the University of Campinas, in the last five years.

**Methods:**

retrospective analysis of medical records of patients classified by levels of injury severity, in 27 children.

**Results:**

only one child underwent surgery for initial failure of conservative treatment (persistent hemodynamic instability), resulting in a 96% overall success rate of the conservative treatment. Five other children (22%) developed late complications that required elective surgery: a bladder injury, two cases of infected perirenal collections (secondary to injury of renal collecting system), a pancreatic pseudocyst and a splenic cyst. Resolution of the complications was attained in all children, with anatomical and functional preservation of the affected organ. There were no deaths in this series.

**Conclusion:**

the conservative initial approach in the treatment of blunt abdominal trauma was effective and safe with high resolution and low rate of complications leading to a high preservation rate of the affected organs. Level of evidence III - prognostic and therapeutic study.

## RESUMO

**Introdução:**

no Brasil, o trauma é responsável por 40% dos óbitos na faixa etária entre 5 e 9 anos, e 18% entre 1 e 4 anos, e o sangramento é a principal causa de prevenção morte na criança traumatizada. O manejo conservador de trauma abdominal contuso com lesão de órgãos sólidos - iniciado na década de 60 - é a tendência mundial atual, com estudos mostrando taxas de sobrevivência acima de 90%. O objetivo do presente trabalho foi avaliar a eficácia e segurança do tratamento conservador em crianças com trauma abdominal contuso tratado no Hospital das Clínicas da Universidade de Campinas, nos últimos cinco anos.

**Métodos:**

análise retrospectiva de prontuários de pacientes classificados por níveis de gravidade da lesão, em 27 crianças.

**Resultados:**

apenas uma criança foi submetida a cirurgia por falha inicial do tratamento conservador (instabilidade hemodinâmica persistente), resultando em uma taxa de sucesso global de 96% do tratamento conservador inicial. Outras cinco crianças (22%) desenvolveram complicações tardias que exigiram cirurgias eletivas: lesão na bexiga, dois casos de coleção perirenal infectada (secundária à lesão de sistema de coleta renal), um pseudocisto pancreático e um cisto esplênico. Resolução da complicação foi atingida em todas as crianças, com preservação anatômica e funcional do órgão afetado. Não houve mortes nesta série.

**Conclusão:**

a abordagem inicial conservadora no tratamento de trauma abdominal contundente foi eficaz e segura com alta resolução e baixa taxa de complicações levando a uma alta taxa de preservação dos órgãos afetados. Nível de evidência III - estudo prognóstico e terapêutico.

## INTRODUCTION

Trauma is a major cause of morbidity and mortality in children and adolescents worldwide, accounting for 10-15% of pediatric admissions in hospitals and intensive care units^1^ ^-^ ^3^. According to data from the Health Ministry of 1995, accidents and violence are the leading cause of death in children and adolescents in Brazil, with 57% of total mortality in children 0-19 years^4^. More recent data show that, in Brazil, trauma accounts for 40% of deaths in the age group between 5-9 years old and for 18% between 1-4 years old^5^ ^,^ ^6^.

Hemorrhagic shock induces hemodynamic disorders but, the higher compensation capacity to ensure perfusion of vital organs, the proportionally largest volume of circulating blood and higher cardiac output are some of the important physiological advantages of children compared to adults that may protect them in the early post traumatic period^1^ ^,^ ^7^ ^,^ ^8^. However, the lack of diagnosis and treatment of internal bleeding are the leading cause of preventable death in traumatized children and usually occur within the first 4 hours after trauma^9^ ^,^ ^10^.

Until 20 years ago, it was believed that surgical treatment should be instituted, as early as possible, in order to reduce morbidity and mortality of trauma. Currently, the global trend is towards the conservative treatment of blunt abdominal trauma, often with prolonged hospitalization and aggressive hemodynamic monitoring of affected children, as well as the intense use of imaging methods to follow-up these children^11^ ^-^ ^15^.

The aim of this study is to evaluate the efficacy and safety of conservative treatment in children with blunt abdominal trauma, treated at the Clinical Hospital of the State University of Campinas Medical School, São Paulo.

## METHODS

This study was approved by the local Ethics Committee (CAAE: 49317121.0.0000.5404). A retrospective, descriptive, cross-sectional cohort study based on an analysis of medical records was conducted including all children under 14 years of age seen during the 5-year period, from 2011 to 2015, with a diagnosis of visceral injury secondary to blunt abdominal trauma. Data collected included age, sex, mechanism of trauma, Glasgow scale, severity - according to ISS scale (Injury Severity Score) and PTS (Pediatric Trauma Score), associated injuries, treatment instituted, preservation or not of the affected organ, complications and final outcomes.

For the classification of intra-abdominal organ lesions, we used the “Injury Severity Scale” of the American Society of Trauma Surgery, for all patients, based on tomographic findings and descriptions of records^16^ ^,^ ^17^.

### Patients

Inclusion criteria: Every patient under 14 years of age, with a diagnosis of visceral injury secondary to blunt abdominal trauma, seen by the Pediatric Surgery team at the Clinics Hospital of the State University of Campinas Medical School between January 2011 and December 2015. No exclusion criteria were applied. A total of 27 patients were included. in the study.

### Management protocol

Over the last thirty years, polytrauma pediatric patients admitted to the Emergency Room (ER) of the Clinics Hospital of the State University of Campinas Medical School are initially evaluated by the emergency pediatrician. The hospital is a tertiary facility, exclusively dedicated to assist patients from the National Health System, that serves as a referral center to a surrounding area of approximately 5 million inhabitants. The pediatric surgery team is on call, does not participate from the initial management and is summoned only when there is evidence or suspicion of visceral injury. Every patient considered as being hemodynamically stable after initial evaluation and stabilization enters the protocol of conservative management. This includes securing of a vascular access, ultrasound and tomographic evaluation of the abdomen, and close observation in the intensive care unit (ICU). Decision to withhold conservative management and proceed to surgery is taken when there is perforation of hollow viscera (digestive or urinary) and/or because of persistent uncontrolled bleeding resulting in hemodynamic instability. Otherwise, the patient is monitored in the ICU for 48 hours and discharged to the ward where he is managed according to the type and severity of the injury. Eventual complications are treated as needed. Bed restriction, resuming of oral feedings and discharge from the hospital, as well as the outpatient follow-up schedule are decided on a case to case basis.

### Statistical methods

Being a retrospective observational study no a-priori sample size was calculated. Descriptive statistics included median and range of numerical data and frequency percentual distribution of descriptive data.

## RESULTS

Data from the 27 patients included in the study are summarized in Table 1. Age ranged from 1-13 years, with a median of 10 years. Only 2 patients were younger than 2 years. There was a male to female ratio of 3,5:1. Fall from owns height and bicycle fall were the most frequent causes of abdominal trauma, accounting for almost 30% of cases each. Three children (11,1%) were involved in car accidents as passengers while 8 (29,6%) were hit by a car.

**Table 1:** All patients of this study and their injuries.

Age (years)	Gender (male / female)	Mechanism of trauma	Pts	Iss	Initial glasgow	Abdominal injury	Associated injury	Surgery	Days between trauma x surgery	Complication	Affected organ preservation
13	M	Direct trauma	12	16	15	Kidney injury - gii	No	No	_	_	Yes
12	F	Run over	7	24	12	Hepatic laceration - gi; splenic laceration - gi; haematoma in rigth adrenal gland	Tbi; pulmonary contusion	No	_	_	Yes
4	M	Run over	11	34	14	Kidney injury - giv (with urinary extravasation); hepatic laceration - gi	Pulmonary contusion; right rib fracture (10th)	Yes	9	_	Yes, partially
8	M	Bicycle fall	12	25	15	Distal transection of pancreas - g iii	No	Yes	300	Pancreas pseudocyst	Yes
13	M	Bicycle fall	12	9	15	Kidney injury - gi	No	No	_	_	Yes
8	M	Run over	12	17	15	Hepatic contusion - giii; haematoma in right adrenal gland	No	No	_	_	Yes
10	M	Bicycle fall	12	16	15	Splenic laceration - giv	No	Yes	505	Splenic cyst	Yes
13	F	Car accident	12	16	15	Hepatic laceration from segment iva to the hilum - giii	No	No	_	_	Yes
1	F	Run over	0	25	ETI	Hepatic laceration (segments vi and vii) - giii; right adrenal gland haematoma	Pulmonary contusion; right rib fracture (1st to 8th); right pneumothorax	No	_	_	Yes
1	M	Run over	3	35	12	Hepatic laceration - giv; major pancreatic laceration with ductal injury - giii; splenic laceration - gi; right adrenal gland haematoma	Bilateral hematothorax; right rib fracture	No	_	_	Yes
Age (years)	Gender (male / female)	Mechanism of trauma	Pts	Iss	Initial glasgow	Abdominal injury	Associated injury	Surgery	Days between trauma x surgery	Complication	Affected organ preservation
9	M	Bicycle fall	11	25	15	Splenic laceration and haematoma - giii	Left hematothorax	No	_	_	Yes
13	M	Bicycle fall	11	21	15	Distal pancreatic laceration - giii; splenic laceration - giii; hepatic laceration (segments i and ii) - gi	Left frontal haematoma; left apex orbital, sinus and frontal fracture	No	_	_	Yes
4	F	Fall from own height	11	16	15	Splenic parenchimal laceration - giii	Portal hypertention with splenomegaly and thrombocytopenia	No	_	_	Yes
13	M	Fall from own height	12	16	15	Splenic laceration - giv	No	No	_	_	Yes
6	F	Car accident	10	20	15	Hepatic laceration (segments vii and viii) - gii	Mild tbi; legs haematomas	No	_	_	Yes
7	M	Bicycle fall	12	17	15	Kidney injury - giii	Mild tbi	No	_	_	Yes
13	M	Bicycle fall	12	9	15	Pancreatis haematoma - gii	No	No	_	_	Yes
5	M	Run over	4	41	9	Splenic laceration - giii	Severe tbi; pulmonary contusion; left pubis, tibia and fibula fracture	No	_	_	Yes
4	F	Fall from a height	12	16	15	Bladder intraperitoneal rupture - giv	No	Yes	1	_	Yes
9	M	Run over	7	26	15	Bladder extratraperitoneal rupture - gii	Pelvis fracture	No	_	_	Yes
11	M	Fall from own height	12	9	15	Splenic laceration - gii	No	No	_	_	Yes
12	F	Car accident	12	25	15	Kidney injury - g iv, to the hilum, with blush and partial exclusion of the kidney, with colector system injury	No	No	_	_	Yes, partially
Age (years)	Gender (male / female)	Mechanism of trauma	Pts	Iss	Initial glasgow	Abdominal injury	Associated injury	Surgery	Days between trauma x surgery	Complication	Affected organ preservation
6	M	Run over	8	22	15	Duodenum and retroperitoneal haematoma - gii	Fracture and iliac - sacral dislocation + severe laceration of right leg	No	_	_	Yes
12	M	Bicycle fall	12	16	15	Hepatic laceration - giii; splenic laceration - g i	No	No	_	_	Yes
9	M	Heavy object falling on abdomen	11	25	15	Hepatic laceration (segments iv and v) - giv	No	Yes	<1 (4 Hours)	_	Yes
11	M	Fall from a height	12	16	15	Left kidney laceration - gi	No	No	_	_	Yes
10	M	Fall from a height	1	43	ETI	Hepatic laceration (segments vi and vii) - giii; splenic laceration - giii; right kidney injury (with urinary extravasation) - giv; left kidney injury without artery contrast - gv	Pulmonary contusion; five lumbar fractures with spinal cord injury	Yes	17	_	Yes, partially

The Glasgow score at the entrance ranged from 9-15 in 25 patients, In the remaining 2 it could not be evaluated because of sedation. 

Associated injuries were present in 12 patients (44%): 5 patients (18,5%) had Traumatic Brain Injury that was graded as severe in one; 5 (18.5%) had pulmonary contusion, with rib fractures in 3 of those; 3 patients (11.1%) had pelvic fractures, 2 (7.4%) had leg fractures and 1 patient (3,7%) had a lumbar vertebrae fracture with spinal cord injury.

Three patients presented with severe shock at admission, which was controlled in two cases. One patient (with a grade IV liver injury) had persistent hemodynamic impairment despite repeated fluid resuscitation and was taken to the operating room 4 hours after admission. This was considered the only failure of initial conservative treatment.

Spleen and liver were the most affected organs. 

### Description of injuries

- 11 patients had liver damage, 3 grade I, one grade II, 5 grade III and 2 grade IV.
- 11 patients had splenic injury, ranging from grade I to IV, that were associated with liver damage in 5 cases.
- 4 patients had pancreatic injury, with duct injury in two of them.
- One child had retroperitoneal and duodenum hematoma, grade II.
- There were no cases of intestinal perforation.
- Genitourinary tract injuries were present in 9 patients:
- 7 kidney lesions (three grade IV injury, with collecting system injury)
- 2 bladder injuries - one intra and other extra peritoneal. The patient with extra peritoneal injury was treated solely by bladder catheterization.

### Need for operative treatment

Upon admission - 2 children:

- The child with intraperitoneal bladder injury underwent laparoscopic bladder suture.
- A child with grade IV hepatic injury required surgical intervention due to persistent hemodynamic instability: at laparotomy there was extensive damage to liver segments IV and V that were treated by suture of the median supra-hepatic vein and hepatorraphy.

### Late operations

- A child with splenic trauma developed a persistent large splenic cyst. Because of persistent pain and also family concerns about cyst rupture during normal sporting activities, it was decided to resect it, which was done uneventfully by laparoscopy.
- A patient with pancreatic avulsion developed a persistent pancreatic pseudocyst that required intragastric diversion in a second hospitalization approximately 3 months from the initial admission.
- Two of the 3 children with renal injury required surgical intervention during the initial hospitalization - one for late correction of a proximal ureteral avulsion and another to drain a perirenal collection.

Overall, 6 out of the 27 patients required surgical intervention to treat an injury related to the abdominal trauma (22.2%). The child with intraperitoneal bladder injury was initially allocated for non-operative treatment because he was stable but, as soon as the diagnosis of perforation of hollow viscera was suspected and confirmed, he was taken to the operating room and submitted to laparoscopic repair of a bladder rupture. Thus, only one child was operated initially because of persistent hemodynamic instability despite vigorous fluid replacement (1 out of 26 or 3.7%). As mentioned above, 4 additional patients needed surgery for treatment of late complications of the initial injury. It is important to notice that these late complications were managed by relatively simple operations that evolved uneventfully and resulted in complete preservation of the organs involved.

Median PTS was 12 with only 5 patients having scores <8. As for the ISS there was an apparent correlation with the need for operation, although not statistically significant: none of the three that were classified as mild trauma (ISS from 1-15) needed surgery; however, 14,2% of the 14 classified as moderate trauma (ISS 16-24) and, 40% of the 10 included as severe trauma (ISS equal or above 25) required surgery.

The present series showed that the initial conservative treatment of blunt abdominal trauma had an efficacy of 96%.

## DISCUSSION

The first report of conservative management of blunt abdominal trauma came from Toronto, between 1956-1965, of 12 children with splenic trauma treated without surgery. Revision of published series showed that 8-12% of children with blunt abdominal trauma have an internal organ damage and more than 90% of them survive^9^ ^,^ ^12^ ^,^ ^18^ ^-^ ^21^.

More flexible ribs, thinner abdominal wall, smaller size of the abdomen and a higher relative volume of the parenchymal organs predispose to the occurrence of multiple injuries in children. However, the thicker and resilient capsule of organs also cause bleeding to cease spontaneously more often, facilitating the non-operative treatment of those injuries^7^.

A few factors may increase the risk of failure in non-surgical treatment: bicycle trauma, isolated pancreatic injury, more than one solid organ or isolated 5th degree lesions of any solid organ and traumatic brain injury patients with Glasgow less or equal to 8. These patients should be carefully evaluated, because conservative treatment failure may have serious consequences^22^ ^-^ ^25^.

Some complications of the conservative treatment are:

- Late hemorrhage, occurring up to 10 days after trauma. Usually manifests as persistent pain or signs of peritoneal irritation (such as pain in the right shoulder), requiring a longer restriction of physical activities^26^ ^,^ ^27^.
- Pseudocysts or splenic pseudoaneurysm - may require laparoscopic excision or marsupialization; embolization for pseudoaneurysm is not widely accepted because there appears to be associated with increased risk of rupture^14^ ^,^ ^28^.
- Failure to identify a hollow viscus injury, like a terminal ileal or duodenal perforation. As initial abdominal radiographs may not detect early retro pneumoperitoneum, these lesions may go undetected in the early phases of follow-up. They should be suspected in children with persistent abdominal pain or vomiting for more of 48hs after the trauma^29^ ^,^ ^30^.

Trauma’s consequences reach far beyond the financial costs, causing emotional, behavioral and child development disorders, making the management of these patients a great challenge to health services.

Liver and spleen were the most affected organs, corresponding to one third of injuries. The conservative treatment of isolated lesions of one of these two organs in stable children is universally accepted and considered standard^31^ ^-^ ^33^. 

Between 1995-1997, American Pediatric Surgical Association created a protocol for ICU treatment for isolated splenic or liver trauma from grade I to IV, based on data from 832 children. And in 1998-2000, the same protocol was applied to 312 clinically stable children, grouped by the severity of injury. Four months after the trauma, none of the stable children required surgery and there was a decrease in length of hospitalization in ICU and removal activities^14^ ^,^ ^33^.

The success of conservative treatment of isolated splenic and liver injuries is higher than 90%. However, Mooney and Forbes^19^ revised the trauma data from the 90’s in England and found that among 2500 children with splenic injury, 2/3 were not treated by a pediatric surgeon or haven’t been treated in a trauma center. On the other hand, Mooney and Ruthstein^34^ found that these children have 2.6 to 2.8 times more chance of undergoing surgical treatment than those treated in an independent pediatric hospital. 

In our series of patients, the conservative treatment was initially adopted in all hepatic and splenic injuries, regardless the degree of involvement of the organ or the presence of associated injuries. The decision to operate was solely based on the hemodynamic status of the patient. In a patient with grade IV liver injury, the hemodynamic instability persisted despite aggressive fluid replacement and surgical intervention was necessary. Among the splenic injuries, there were no failures of conservative treatment. However, one child developed a persistent splenic cyst that needed surgery which was done in a subsequent hospitalization. It is believed that a scheduled operation to treat late complications, with a refreshed and prepared surgical and anesthetic team, is safer than emergency surgery, which often occurs in the middle of the night, in a unstable and actively bleeding child.

Another study carried out in adults from our hospital, considered conservative treatment of splenic injuries grade IV to be safe, failing in only two patients (7.7%), that were operated due to worsening of abdominal pain and hypovolemic shock; there were no complications or deaths^22^. Another Brazilian study, concluded that ISS and grade of splenic injury have direct and significant relationship with the failure rate of the non-operative treatment, a relationship that was not confirmed in the present study^35^. The fact that in our service, the decision to operate does not take in account the grade of the injury, may help explain these discrepancies.

As for liver trauma, surgical approach is reserved to cases with refractory shock - only 4% of injuries. Presently, criteria for surgical intervention is roughly defined as the necessity of replacing more than 40% of the blood volume, associated with persistent or recurrent hemodynamic instability^1^ ^,^ ^14^ ^,^ ^27^.

Some surgeons resist non-operative management, even for isolated splenic injury, stating that if no intervention is made, other injuries, especially involving hollow viscus, could go unnoticed. However, Morse and Garcia showed that only 22 (18%) of 120 children undergoing conservative treatment had other associated lesions, and only 3 (2.5%) had gastrointestinal lesions^36^. In the present study, no child had associated gastrointestinal perforation and only one presented a duodenum hematoma. However, as stated before, late presentation of gastrointestinal perforation is a possibility and the medical team should be aware and attentive to make a prompt diagnosis in case this occurs. 

Pancreatic and duodenal lesions are much rarer and occur in less than 10% of patients with blunt abdominal trauma. In the absence of lesion of the pancreatic duct or clinical deterioration of the patient these injuries may be managed with conservative treatment, and 10% will eventually require surgery to drain a pseudocyst. When however, there is evidence of injury to the pancreatic duct a great number of surgeons still prefer to operate raising the operative rate to about 20% of the children^21^ ^,^ ^28^.

In San Diego, Canty and Weinman studied 18 patients with major pancreatic lesions, concluding that the distal lesions should be treated with distal pancreatectomy, with the proximal ones being amenable to observation and, when necessary, pseudocyst drainage; they also found that, in acute phase, ERCP with stent placement is safe and effective^37^ ^,^ ^38^. 

In the present study, conservative treatment of pancreatic trauma was considered satisfactory - only one of four pancreatic trauma patients required internal drainage of a persistent pseudocyst secondary to total transection of the duct. Although the treatment of pancreatic injury remains controversial, these data show that it is amenable to initial conservative treatment, even when there is injury of the pancreatic duct.

In blunt abdominal trauma, the clinical signs of urological involvement are unspecific. Therefore, it is essential to suspect the injury by evaluating the mechanism and the forces involved in trauma. The kidney is more commonly injured in children (approximately 10% of abdominal blunt trauma) than in adults, due to less protection from perirenal fat and a lower position of the kidney in this age^39^ ^,^ ^40^. Renal injury associated with other lesions occur in up to 74% of patients with abdominal trauma - often involving the liver and spleen^8^. Children seem to have a greater capacity of functional recovery than adults^41^. In 98% of cases, renal injuries can be treated conservatively^42^ ^,^ ^43^.

Absolute indications for surgical treatment of renal injury, for some authors, are refractory hemodynamic instability and the presence of other associated injuries. Even in cases of large leakages of urine, surgical indication is controversial with spontaneous resolution rates over 80% of cases^43^. Lesions grade IV and V occur in only 5% of renal trauma and possibly require surgical treatment but, in selected patients, even these high-grade lesions can be initially treated conservatively^42^ ^-^ ^44^.

Nephrectomy rates after immediate exploration vary considerably depending on the type and degree of injury reaching close to 100% in unstable patients with severe injuries. However, with the improvement of imaging exams, the need for early surgical exploration decreased and renal preservation rates increased^39^.

In this study, we found kidney injuries in 6/27 patients (22%), and 2 of them had associated injuries. Conservative treatment was first instituted in all six, but two required late surgical treatment during the same hospital stay for resolution of a persistent urinary fistula. Complete anatomical and functional preservation of the kidneys was achieved in 3 cases. In the remaining 3 cases (two who were operated and one treated conservatively) there was partial loss of renal function, probably due to the extensive trauma.

Ureteral injury by blunt trauma is extremely rare and occurs primarily in children with urinary tract congenital abnormalities, and when the diagnosis is made early, immediate surgical correction appears to be the most appropriate action^40^.

In one of the cases of renal trauma a delayed diagnosis of ureter avulsion at the ureteropelvic junction was made by ascending pyelography. This patient underwent initial drainage of the perirenal collection and, after a week, a definitive procedure to reconstruct the ureter avulsion. According to other authors this type of lesion is often diagnosed late in the course of the treatment of renal blunt trauma and usually its surgical treatment is postponed, since interventions during this period are considered to be more difficult because of intense inflammatory process, which predisposes to greater damage to the ureter, restricting the initial treatment to symptom relief and preservation of renal parenchyma with temporary urinary derivations^40^ ^,^ ^45^. 

Bladder lesions are classically divided into intra and extraperitoneal. In the extraperitoneal lesions, the classical treatment is conservative, with indwelling catheter for 10 days, associated with antibiotic therapy, with resolution of about 85% of the lesions, confirmed by cystography, at the time of the Foley catheter removal^40^. This protocol was applied successfully to the only case of extraperitoneal bladder injury in the present series.

One child presented with bladder dome rupture and was operated as soon as the diagnosis was established. In intraperitoneal bladder injuries, the laceration is generally in the dome, which is the most fragile region of this organ. And, the classic treatment for intraperitoneal bladder rupture is surgery^46^. 

Whenever surgery is required, laparoscopy must be considered. It allows early assessment of all the cavity, peritoneum wash, cauterization and sutures, with rapid recovery and less chance of nosocomial infection. However, its use in abdominal trauma remains limited especially in local hospitals because of high cost, need for special equipment and trained personnel and risk of air embolism or hypertensive pneumothorax by pneumoperitoneum. In cases of hemodynamic instability and diaphragmatic rupture, for instance, laparoscopic surgery and artificial pneumoperitoneum are contraindicated^47^.

In a study of 33 children victims of trauma and hemodynamically stable undergoing laparoscopy, conversion to laparotomy occurred in 8 cases, due to intense uncontrolled bleeding^2^.

Of the six children operated in this series, 3 were initially approached through laparoscopy but, in two, it was converted to open surgery, due to technical difficulties; two other patients were operated through a small laparotomy and in the one unstable patient with liver laceration, emergency midline laparotomy was the incision of choice.

Trauma severity indexes have several functions: quantify injuries and anatomical and physiological changes (trauma`s magnitude); determine survival prognosis; serve as a basis for screening in accidents with multiple victims or disaster; establish lines of clinical research and epidemiology; assess and monitor the quality of care for traumatized and allow the implementation of prevention of accidents and violence programs. They may also be used for the comparison of mortality between groups of patients with similar severity of trauma, and the assistance provided and the effectiveness of the measures imposed as well^17^.

The Pediatric Trauma Score (PTS), uniquely developed for dimensioning traumatic injuries in children consists of six parameters each with three possible scores: patient’s size, airways, consciousness, systolic blood pressure, the presence or absence of fractures and skin lesions. This scale has been shown to be useful as an index of severity predictor, especially assessing the risk of early mortality. A significant mortality risk is expected when this score is <8^48^ ^,^ ^49^. In our series only 6 patients had scores <8.

In the study of Gennari & Koizumi, the mortality rate in the closed group of trauma patients with ISS from 1-15 was 1%; 12.5% in the group with ISS 16-24 and, from 25, there was almost linearly increased mortality^50^.

In the present series, despite the fact that almost 45% of the children presented with associated injuries, there was no mortality, not even in the group with ISS >25. It may be that this low mortality rate is a result of improvements in trauma life support, both in the pre-hospital care as well as in the reference services and intensive care units. Another possible explanation is that, because ours is a tertiary referral hospital, situated in a non-central area, eventual patients with severe life-threatening extra-abdominal injuries, might have died from these injuries in the initial emergency facility, before even being referred to our hospital. 

It should be stressed out that one of the key points of the non-operative treatment of abdominal trauma is the 24 by 7 availability of a medical team (pediatric surgeons, anesthesiologists, radiologists and pediatric intensivists) prepared to detect and treat any eventual complications that may occur^51^ ^,^ ^52^. Safety of this approach can only be guaranteed in first-line trauma centers capable of treating severely injured and highly complex patients, offering them the possibility of definitive treatment of traumatic injuries^53^.

In the present series, fall from owns height and bicycle fall were the most frequent causes of abdominal trauma, accounting for almost 30% of cases each. Three children (11,1%) were involved in car accidents as passengers while 8 (29,6%) were hit by a car. Reports from different institutions report different epidemiological distributions of the causes of trauma, reflecting the differences in the type and localization of the hospitals^3^ ^,^ ^6^ ^,^ ^18^ ^,^ ^21^. In the present series the main causes of trauma are in accordance with previous data from our institution. A review of the causes of traumatic deaths in children and adolescents revealed that among 530 trauma-related deaths occurring between 2001 and 2008 there were 138 road traffic related deaths of which 44 were passengers and 77 were pedestrian victims^54^.

## CONCLUSION

The presented series indicates that, in stable children with blunt abdominal trauma, initial conservative approach was effective and safe with a high resolution of injuries and low rate of complications resulting in a high index of preservation of the affected organs. The selective treatment of eventual late complications instead of an initial operative approach has avoided 21 unnecessary operations and resulted in sparing 100% of the organs afflicted.

**List of abbreviations**
CT : = Computed Tomography
ER : = Emergency Room
ERCP : = Endoscopic Retrograde Cholangiopancreatography
ICU : = Intensive Care Unity
ISS : = Injury Severity Score
PTS : = Pediatric Trauma Score
